# Mesenchymal Stromal Cells in Osteoarthritis: Evidence for Structural Benefit and Cartilage Repair

**DOI:** 10.3390/biomedicines10061278

**Published:** 2022-05-30

**Authors:** Yujie Song, Christian Jorgensen

**Affiliations:** IRMB, CARTIGEN, University of Montpellier, Inserm, CHU de Montpellier, 34090 Montpellier, France; yujie.song@inserm.fr

**Keywords:** cell therapy, mesenchymal stromal cells, adipose tissue, bone marrow, osteoarthritis, MRI

## Abstract

Osteoarthritis (OA) presents a major clinical challenge to rheumatologists and orthopedists due to the lack of available drugs reducing structural degradation. Mesenchymal stromal cells (MSCs) may represent new therapeutic approaches in cartilage regeneration. In this review, we highlight the latest knowledge on the biological properties of MSC, such as their chondrogenic and immunomodulatory potential, and we give a brief overview of the effects of MSCs in preclinical and clinical studies of OA treatment and also compare different MSC sources, with the adipose tissue-derived MSCs being promising. Then, we focus on their structural benefit in treating OA and summarize the current evidence for the assessment of cartilage in OA according to magnetic resonance imaging (MRI) and second-look arthroscopy after MSC therapy. Finally, this review provides a brief perspective on enhancing the activity of MSCs.

## 1. Introduction

Osteoarthritis (OA) is the most common and chronic form joint disease, predominantly characterized by synovial inflammation and cartilage degradation leading to joint space narrowing associated with subchondral bone lesions, and these alterations can be visualized on X ray and magnetic resonance imaging (MRI). OA is a major cause of pain and disability affecting the ageing population with increasing prevalence as this population expands [1,2]. It is expected that by 2030, 20% of adults will have developed OA in Western Europe and North America [3], which imposes the morbidity and economic burdens on patients, their families and the society [4]. Notably, recent studies have also showed robust evidence that OA is a significant risk factor for cardiovascular diseases (CVD) and suggested that patients with OA are twice as likely to develop ischemic heart disease and heart failure which increase mortality [5,6].

Currently, disease modifying treatments of OA are limited, whose goal is to relieve pain and improve function by controlling inflammation, but have little impact on the progressive degeneration of articular cartilage. Thus, efficient therapeutic methods are urgently needed to be explored for cartilage restoration. Consequently, regenerative medicine may overcome existing barriers and develop a superior treatment to alleviate OA or rheumatic diseases as a whole.

Among various types of stem cell, mesenchymal stromal cells (MSCs) are one of the most common stem cells that are used in cell therapy. MSCs are strongly immunosuppressive cells, which can migrate to injury sites, induce peripheral tolerance, inhibit the release of pro-inflammatory, and decrease monocyte activation. They can also promote tissue repair and the survival of damaged cells [7]. Previous clinical trials have demonstrated that OA can be treated efficiently using autologous or allogenic MSCs through implantation, microfracture, or intra-articular (IA) injections [8,9,10,11]. Despite improvement in pain are continually reported, there is a need to confirm the long-term effect on cartilage tissue. 

Therefore, this review summarizes the available evidence of MSC therapy as a promising strategy for OA. Here, we focus on the valuable biological characteristics of MSCs for treating OA, then highlight the clinical applications of MSCs as well as explore the assessment of cartilage in OA based on the results of MRI and second-look arthroscopy after MSC therapy. Last but not the least, we present our perspectives on enhancing the activity of MSCs.

## 2. Biological Properties of MSCs

MSCs can be derived from bone marrow, adipose tissue, umbilical cord blood, skin, and other sources [12], but bone marrow MSCs (BM-MSCs) and adipose-derived stem cells (AD-MSCs) are the most commonly used MSCs for cartilage repair because of their relatively easy availability and wide ethical acceptability [13]. There are several biological similarities and differences between both BM-MSCs and AD-MSCs. For example, both of them showed similar adhesion and proliferation on nanoparticle-coated plates [14] and displayed a highly similar morphology and marker expression in an undifferentiated state [15], but compared to AD-MSCs, BM-MSCs with or without scaffolds exhibited an enhanced capability to differentiate into the chondrogenic lineage [15,16]. Further, concerning their chondrogenic potential, AD-MSCs had statistically weaker chondrogenic potential with regard to matrix formation and cell morphology compared to BM-MSCs [17]. The chondrogenic differentiation of BM-MSCs was observed by the formation of extracellular matrix (ECM), as well as the synthesis of type II collagen and proteoglycans. The main challenge of chondrogenic induction of BM-MSCs is to control their differentiation, as BM-MSCs tend to present a hypertrophic phenotype that leads to calcification [18], while in order to achieve desirable chondrogenic differentiation of AD-MSCs, it was suggested to use of higher concentrations of specific growth factors [19] or develop three-dimensional scaffolds that would overcome the growth inhibition and support chondrogenesis [20].

On the other hand, in a recent study which compared the differences of AD-MSCs and BM-MSCs at single- and bulk-cell levels on the treatment of OA, it was found that AD-MSCs were a more stable and controllable source of stem cells, better adapted to survive in the hypoxic articular cavity niche, and demonstrated superiority in regulating inflammation [21]. AD-MSCs can maintain their phenotype longer in culture, display in a higher proportion (5% compared to 0.01%) in the source tissue, and can be obtained by a minimally invasive and painless procedure compared with BM-MSCs [22,23]. Moreover, AD-MSCs can be transplanted to autologous or allogenic bodies safely with less implant migration and foreign body reaction [24]. AD-MSCs can also differentiate into cell types of the three developmental germ layers (ectoderm, endoderm, and mesoderm), which include adipogenic, osteogenic, chondrogenic, myogenic, angiogenic, cardio-myogenic, tenogenic, and periodontogenic lineages [23,25,26,27,28,29,30,31,32,33]. It has been shown that AD-MSCs facilitate tissue regeneration and repair by secreting growth factors, cytokines, angiogenic factors, adipokines and neurotrophic factors to stimulate restoration of normal tissue function or reduce damage [34]. The cytokine profile of AD-MSCs consists of pro/anti-inflammatory, angiogenic and hematopoietic factors, being interleukins (IL-6, IL-7, IL-10, IL-11), vascular endothelial growth factor, fibroblastic growth factor, tumor necrosis factor-alpha, and granulocyte or macrophage colony-stimulating factor [35]. Molecules released by AD-MSCs include growth factors, enzymes, such as tryptophane kinase, TIMS, and extracellular vesicles. These paracrine factors play positive roles in the general vitality of cells and mechanisms involved in the central nervous system, immune system, heart, and muscles [36]. Additionally, AD-MSCs are found to express genes and proteins for cartilage-specific molecules, including type II collagen and aggrecan, but lack expression of hypertrophic chondrocyte markers, such as type X collagen [37,38,39]. Furthermore, the immunosuppressive properties of AD-MSCs can be the result of releasing prostaglandin E2 and increasing indoleamine 2,3-dioxygenase (IDO) [40]. The immunophenotype of AD-MSCs can express other essential factors participating in stemness, self-renewal, and differentiation potentials, such as CD146 and CD271. They are also associated with enhanced healing capacity of bone defects or cartilage, or with differential paracrine wound healing activity [41,42]. 

## 3. Structural Benefits to MSCs in OA Therapy

### 3.1. Preclinical Evidence

Over the past decade, there have been ongoing efforts in preclinical evaluation of MSCs for the treatment of OA. It has involved a wide variety of animal models, ranging from small to large animals, different induction methods for OA model, MSCs origins, and administration regimes.

Different OA models were performed with allogenic or autologous BM-MSCs or AD-MSCs, as well as umbilical cord-derived MSCs (UC-MSCs), etc. Similar clinical improvements from 8.5 to 26 weeks between single and repeated injection with allogenic UC-MSCs were observed in horses OA [43]. It is argued that UC-MSCs may have greater biological potential and an immunologically privileged state compared to adult MSCs [44]. While in a rat OA model, it was demonstrated that a single injection of UC-MSCs could have temporary effects (2 weeks) on decelerating the progression of cartilage degeneration, but may not inhibit OA progression in the long-term (8 weeks) [45]. In another study, after a weekly repeated IA injection for three weeks, UC-MSCs prevented cartilage degradation, restored the proliferation of chondrocytes, and inhibited the inflammatory response in rats OA [46]. UC-MSC transplantation in a rabbit OA model showed a significant decrease in the expression of MMP13 in cartilage and improvement in the joints on histological findings, such as less hyaline cartilage destruction and higher ICRS histological score. However, the MRI study could only detect the improvement in 6 weeks, but not in 12 weeks [47]. On the other hand, A study reported that benefit with less cartilage fibrillation and subchondral bone sclerosis and increased lubricin was found at 26 weeks with a single injection of 20 million allogenic AD-MSCs with high expression of α10-β1 integrin in the equine OA [48]. Similarly, enhanced synovial outcome of 5 million autologous AD-MSCs with single injection has been observed in naturally occurring OA equine model at 13 weeks [49]. The benefit of AD-MSCs injection were associated with the secretion of anti-inflammatory factors, including hepatocyte growth factor, human leukocyte antigen G5, or interleukin-1 receptor antagonist [50]. In another study, 18 healthy horses underwent a chemically induced procedure to create OA, then divided into three groups depending on the treatment injected: BM-MSC-naïve (*n* = 7), BM-MSCs-primed (*n* = 7), and control (*n* = 4). Beneficial effects of repeated IA administration of allogenic BM-MSCs were mainly found in limiting inflammation and subsequent cartilage degradation in an early stage (8.5 weeks), and suggested higher anti-inflammatory and regulatory effect by primed BM-MSCs treatment [51]. More recently, BM-MSCs cultured under physioxia displayed a significant improvement in cartilage repair score and greater cell numbers with enhanced chondrogenic differentiation potential in focal early OA defect rabbits. However, there remains an open question concerning whether physioxic MSCs are an appropriate cell type for cartilage regeneration [52]. The authors also argued that focal OA models only induce a mild degeneration in the joint, which meet the minimal criteria for early OA, such as impaction or groove models [52]. 

In addition to tissue-derived MSCs, human pluripotent stem cells (hPSCs) have prompted the prospects and feasibility of alternative MSC generation for regenerative medicine. Zhang et al. demonstrated that the cell population after hPSC-MSC therapy exhibited preferable restorative and ameliorative function on OA rabbits [53]. As hPSC-MSCs have various advantages, including the infinite proliferation potential, no ethical risks, homogeneity and illimitation in supply, they could be a potential MSC origin for OA treatment. 

### 3.2. Clinical Evidence

With regard to clinical applications, in a case series of 9 OA patients (11 knee) with 18-month follow up, it was found that the single IA injection of 5–10 million autologous AD-MSCs was a safe and efficient method for treating OA. At 6-month follow-up, significant improvements were found in the clinical symptoms valuated by Knee Society score (41.4 points), Hospital for Special Surgery knee score (33.9 points), Tegner-Lysholm score (44.8 points) and VAS of pain (from 54.5 to 9.3), and MRI assessment of structural cartilage visualization with MOCART score (from 43 ± 7.2 to 63 ± 17.1), and results at the 12- and 18 month-checkpoints preserved the values achieved at the 6-month evaluation [54]. Nevertheless, in controlled clinical trials, long-term outcomes remained contradictory. A 2-phase clinical trial designed to assess the safety and therapeutic potential of IA injected AD-MSCs in patients with OA: the phase I study consisted of three dose-escalation cohorts: the low-dose (1 × 10^7^), mid-dose (5 × 10^7^), and high-dose (10 × 10^7^) group with 3 patients each; the phase II included 9 patients receiving the high-dose, which showed that IA injection of high-dose AD-MSCs into the osteoarthritic knee improved function (WOMAC, from 54.2 ± 5.2 to 32.8 ± 6.3) and pain (VAS, from 79.6 ± 2.2 to 44.2 ± 6.3) without causing adverse events and reduced cartilage defects by regeneration of hyaline-like articular cartilage at 6-month follow-up [55]. A cohort study including 18 patients with knee OA, were divided into 3-dose groups of autologous AD-MSCs treatment: low dose (0.2 × 10^7^), mild dose (1 × 10^7^), and high dose (5 × 10^7^), and found that patients treated with only low-dose group experienced significant improvements in pain levels (VAS, mean change −41.2 ± 13.3) and function (WOMAC, mean change −35.7 ± 10.5 ) compared with baseline at the 6 months of follow-up [56]. Moreover, another similar trial with the same study design and purpose consisted of 18 patients with dose escalation: low dose (1 × 10^7^), mild dose (2 × 10^7^), and high dose (5 × 10^7^). The results implied that after 24 months of follow-up, the high-dose group exhibited better pain alleviation and greater improvement in knee function than the other two groups with the repeated injection of autologous AD-MSCs [57]. 

Despite all the trials used the same cell source without any adjuvants, the number of injections, the follow-up time, and the definition of low, mid, or high dose varied. Therefore, it is still hard to draw a conclusion for the long-term efficacy of AD-MSCs for OA patients. Although the current literature for the clinical efficacy of AD-MSCs for OA is promising, it remains limited to studies without comparing to other treatments because nowadays the extent and duration of placebo effect in knee OA has become a new factor to consider. Further studies with longer follow-up, larger sample size, and an appropriate control would be necessary for addressing unanswered concerns.

We also summarized the recent meta-analysis to give a general view of clinical evidence on MSCs (Table 1). It was demonstrated that MSCs IA injection (cell infusions from 1 × 10^6^ to 1.5 × 10^8^) was safe and has significant potential as an effective clinical therapy for patients with knee OA during follow-up from 12 to 25.7 months [58]. This study included 582 knee OA patients (mean ages from 32 to 57 years, sample size from 14 to 80). Among them, 444 were treated with BM-MSCs, 94 were injected with AC-MSCs, 49 with peripheral blood derived MSCs, and 14 with synovium-derived MSCs. At the 12-month checkpoint, the MD of WOMAC changes was statistically significant at −11.05, in Lequesne was statistically significant at −5.32, while at the 24-month checkpoint, the mean difference (MD) of changes in VAS of patients receiving MSCs treatment was a significant decrease of −5.78, in IKDC was statistically significant at 4.89, in Lysholm was 7.96, in Tegner was statistically significant at 0.46. Another meta-analysis showed the similar results that MSCs therapy ameliorated the overall outcomes of 565 patients with Knee OA, including pain reduction (VAS) and functional improvement (either IKDC, Lysholm, or WOMAC) from basal evaluations based on the effect size values, particularly at 12 and 24 months after follow-up. It is also worth mentioning that the beneficial effect was maintained for two years after treatment, which is to say, the treatment effectiveness did not reduce over time [59]. Another study demonstrated that MSC treatment alleviated knee pain (SMD: −1.45, 95% CI: −1.94, −0.96) and improved physical function (SMD: 1.50, 95% CI: 1.09, 1.92), as well as cartilage quality (SMD: −1.99; 95% CI: −3.51, −0.47), without any severe adverse events in 2385 patients (56.7 ± 6.78 years, from 36.0 to 74.5 years) with OA during a follow-up period of 3 to 60 months. They identified that the pooled effect size on the VAS pain score exceeded the effects of nonsteroidal anti-inflammatory drugs and corticosteroid injections. The MD after intervention were ≥10% for both pain and function, exceeding the minimum for clinically important differences, and meeting the responder criteria of the Outcome Measures in Rheumatology Clinical Trials and Osteoarthritis Research Society International [60]. In the study with 724 patients (mean age 44.2 years), it was concluded that the administration of non-cultured BM-MSCs can significantly reduce pain and improve knee function during a mean follow-up from 3 to 75 months, and recommended to present the culture conditions to which the BM-MSCs have been exposed, the type and contents of culture media, and the culture duration as well as the number of passages in the further studies, because one of the critical issues to maintain the efficacy, safety, and stemness of BM-MSCs cultured ex vivo is the nature and characteristics of the culture conditions [61]. In the study of Han et al., the improvement of VAS scores was statistically significant after BM-MSCs treatment at 6-, 12-, and 24-month follow-up compared with control groups (*p* < 0.01). In contrast, the improvement of WOMAC and Lysholm scores were of no statistical significance, but showed a positive trend with the prolongation of the follow-up time. However, VAS and WOMAC scores of patients after AD-MSC treatment were significantly improved at any follow-up time (*p* ≤ 0.05), but the improvement of Lysholm scores was of no statistical significance. Therefore, this study of 377 OA patients showed that regeneration with BM-MSCs or AD-MSCs had a great application potential in the treatment of patients with knee OA, and AD-MSCs tended to be superior to BM-MSCs according to statistical comparisons of VAS and WOMAC scores [62]. In a meta-analysis consisting of 461 patients, it was indicated that there was no significant difference in the score change rate of OA between the treatments, but AD-MSC treatment showed considerably less variable outcomes than BM-MSC treatments (AD-MSCs, 52.76 ± 3.60; BM-MSCs, 48.23 ± 5.37); the highest change rate of the BM-MSC group was 79.65%, while the lowest was only 22.57%. Thus, the meta-analysis results indicated that the therapeutic effect of BM-MSCs was more variable, suggesting that AD-MSCs may be a more stable cell source for OA treatment [21].

## 4. Assessments of Cartilage in OA

### 4.1. MRI Evaluation after MSCs Therapy

The size, depth of cartilage defect, and signal intensity of cartilage in OA could be measured using MRI or combined with MOAKS (magnetic resonance imaging osteoarthritis knee score) system or MOCART (magnetic resonance observation of cartilage repair tissue) score. It is also pointed out that MRI quantitative T2 mapping has significantly higher sensitivity (*p* < 0.001) in the detection of cartilage lesions within the knee joint at 3.0 T compared with a routine MRI protocol alone, with the greatest improvement occurring in the identification of early cartilage degeneration. Moreover, T2 relaxation time is sensitive to both changes in cartilage hydration and collagen fibril orientation [63,64], and also is longer in remodeling inflammatory tissue versus hyaline cartilage and increases in OA [65,66].

In a meta-analysis study involving 256 patients (mean age from 53 to 61 years) during a follow-up from 3–12 months, 163 patients received BM-MSCs, 67 treated with AD-MSCs, and 26 injected with UC-MSCs. They were compared with placebo or a control group receiving HA. The authors illustrated that pain (VAS MD: 13.24, 95% CI: −23.28, −3.20) and function (IKDC, MD: 40.75, 95% CI: 34.45, 47.05) were improved in patients with knee OA, but no changes were observed in the WORMS (Whole-Organ Magnetic Resonance Imaging Score) of the MRI, which indicated that there was no evidence cartilage was repaired after MSC IA injection (cell infusions ranging from 3.9 × 106 to 1.5 × 108 cells) [67]. Another systematic review found improvements in pain and function after IA MSCs in OA but also summarized that 6/7 prospective observational included studies that reported improvements in cartilage repair, while 1 study reported improvement on delayed gadolinium-enhanced MRI of cartilage and T1rho in 3 of 6 patients [68]. Moreover, 9 studies reported improvements in cartilage status as measured by MRI, while 2 studies reported little or no improvement [56,69]. Of the 4 comparative studies assessed utilizing MRI, 2 reported notably high WORMS scores for cartilage quality in the MSC group [70,71], while 1 reported no significant difference in WORMS scores [69]. The other study reported improved WORMS scores in all groups at 6 months, which was worsened in the control and low-dose groups but remained unchanged in the high-dose group at 12 months [72].

A study with 53 patients (mean age, 57 years) being randomized and treated with 2 injections of 5 × 10^7^ autologous AD-MSCs showed improvement in WOMAC, VAS, and SF-36 scores at 12-month follow-up compared with baseline. Compared with the HA group, more patients achieved a significant 50% improvement of WOMAC and a trend of more patients achieved a 70% improvement in the AD-MSC group after 12 months. Meanwhile, there was a remarkable increase in articular cartilage volume of both knees in the AD-MSC group than in the HA group after 12 months based on MRI [73].

The MRI evaluation after MSC therapy is controversial, as no current evidence supporting a structural benefit with MSC in patients with OA is available. The previous study presents some biases, such as lack of a control arm, heterogeneity in OA patients’ phenotypes, MRI sensibility, MRI techniques and standardization.

Future studies of MSCs therapy without adjuvant treatments are demanded to accurately assess the efficacy and the endpoint of the structural benefit of MSCs on cartilage repair in OA, via MRI T2 mapping if possible.

### 4.2. Second Look Arthroscopy

Arthroscopy has been performed to evaluate any changes, such as size, in cartilage defect at the time of cell injection and at any follow-up time point under the approval of the ethics committee using International Cartilage Repair Society (ICRS) score system. After arthroscopy, biopsy specimens are subjected to safranin O staining and immunohistochemistry for collagen I and II, or hematoxylin and eosin staining to see if there is hyaline-like cartilage or osteoarthritic chondrocytes. Thickness of cartilage could be measured, and specimens could be evaluated with ICRS score as well.

On second-look arthroscopy, numerous studies found improved cartilage status [11,55,72,74,75,76], and 3 showed hyaline-like cartilage [11,55,76], whereas it was reported that all patients showed signs of severe OA (osteoarthritis research society international histologic grade > 3), and only 1 out of 11 cases was observed with stem cell grafting on the cartilage surface at 3-month follow-up [56]. In another 2 comparative studies, improved ICRS or arthroscopic scores were observed in the MSC group via second-look arthroscopy after 19.8 and 10.5 months, respectively [72,76]. It is worth noting that among all the studies discussed above, Wakitani et al. used autologous BM-MSCs with high tibial osteotomy (HTO) [72], and Koh et al. performed autologous AD-MSCs with HTO and PRP as a concomitant treatment [76]. In the case control study of Kim et al., autologous AD-MSCs were used with PRP in injection and fibrin in surgery in each group [74], While Park et al. used allogenic UC-MSCs with multiple drilling (5 × 5 mm) with a follow-up of 84 months [11].

## 5. Perspectives

The properties of MSCs are impacted by biological, biochemical, and biophysical factors in vivo and in vitro that strongly regulate the function of MSCs and the survival reciprocal interactions between the cells, ECM, and soluble bioactive factors. MSCs interact with surrounding tissues and cells in a 3D space to regulate the ECM, therefore promoting angiogenesis, producing anti-inflammatory molecules, preventing cell death (anti-apoptotic effects), and modulating the immune system [77]. In this regard, the high sensitivity of MSCs to the harsh microenvironment of immune-mediated, inflammatory, and degenerative diseases still limit the success of MSC therapies in clinical practice. Therefore, how to enhance the activity of MSCs, including their survival, proliferation and migration capacity, multilineage differentiation potential, immunosuppressive, immunomodulatory, and regenerative functions, in order to improve their therapeutic efficacy and expand their applications, is a current issue that needs to be addressed.

On one hand, it is shown that the therapeutic potential of MSCs can be mainly attributed to their paracrine factors, particularly extracellular vesicles (EVs). EVs are nano-sized (30–150 nm), secreted by all types of cells, and exist in all bodily fluids, which can transfer a variety of bioactive molecules to damaged cartilage and exert positive modulation. A recent study uncovered that MSC-EVs not only promoted the proliferation and migration of human OA chondrocytes, but also maintained the chondrocyte matrix by increasing type II collagen synthesis and decreasing MMP-1, MMP-3, MMP-13 and ADAMTS-5 expression in the presence of IL-1 in vitro; moreover, IA injection of MSC-EVs significantly attenuated OA progression and protected cartilage from degeneration in both rat and mouse OA models [78]. However, EVs in synovial fluid are also cleared rapidly due to the elimination effect of capillary and lymph vessels in synovia membrane on nanoparticles. Therefore, improving the bioavailability of IA injected MSC-EVs is the high priority for their future clinical application.

On the other hand, exosomes, as one kind of EVs, originate from multivesicular endosomes, are able to maintain viability under extreme conditions, and can even reduce oxidative stress. There is also emerging evidence in vivo demonstrating that MSC derived exosomes moderated OA by enhancing cartilage formation and equilibrating the synthesis and degradation of cartilage ECM [79,80]. Indeed, exosomes showed promising results as a future therapy without cells for OA patients. However, there remain challenges to conquer before putting it into clinical application, such as therapeutic effects of exosomes derived from different sources and the separation methods for exosomes.

## 6. Conclusions

This review shows that MSCs have great potential to reduce pain, improve function, and repair cartilage. However, the crucial challenge in MSCs therapy is to empower their activity by mimicking the natural MSCs niche in vitro culture methods, while at the same time allowing cell expansion at a clinical-grade scale (GMP), not compromising cell quality attributes and function. Although further clinical and fundamental studies are highly required, MSC therapy for OA appears to be promising and exciting.

## Figures and Tables

**Table 1:** Meta-analyses of MSC clinical application for OA.

Author	Meta-Analysis	Studies	n	Results
Cui G et al., 2016 Exp Ther Med. [59]		10 single-arm,	565	MSCs significantly improved pain, function after 12 & 24 months.
	18 studies, Knee OA.	4 quasi-exp studies,		Pooled effect size = 2.03 (95% CI, 1.30–2.76) at 12 months.
		4 RCTs.		No dose-responsive association in the MSC numbers was demonstrated.
Lijima H, 2018 NPJ Regen Med. [60]	35 studies, Knee OA.	21 single-arm,	2385	MSCs improved pain, function.
		7 quasi-exp studies, 7 RCTs.		Autologous MSCs had a larger pain relief effect than those in allogenic MSCs. Performing rehabilitation was significantly associated with better self-reported physical function.
Yubo M et al., 2017 PLoS One. [58]	11 studies, Knee OA	11 RCTs.	582	MSC treatment could significantly decrease VAS and increase IKDC scores after 24 months (*p* < 0.05) & decrease WOMAC and Lequesne scores after 12 months (*p* < 0.01).
Awad M et al., 2019 Stem cells Int. [61]	33 studies, OA as well as cartilage defect:	4 RCTs,	724	BM-MSCs: VAS significantly improved (MD = 4.39, 95% CI: 3.19 to 5.58).
	16 studies, cultured BM-MSCs, 17 studies, non-cultured BM-MSCs.	11 cases series, 7 case reports, 7 observational cohorts, 4 quasi-exp studies.		IKDC function significantly improved (MD = 40.75, 95% CI: 34.45 to 47.05).
Zhou W et al., 2019 Am J Sports Med. [21]	14 studies, OA	5 RCTs, 6 cases series, 3 observational cohorts.	461	AD-MSCs showed considerably less variable outcomes than BM-MSCs (52.76 ± 3.603, 48.23 ± 5.374, respectively); the highest change rate of the BM-MSCs was 79.65%, while the lowest was only 22.57%.
Han X et al., 2020 J Comp Eff Res. [62]	9 studies, Knee OA: AD-MSCs & BM-MSCs based in OA	9 RCTs.	377	BM-MSCs significantly improved VAS at 6,12, and 24 months but not WOMAC (*p* < 0.01). AD-MSCs significantly improved VAS and WOMAC scores at any follow-up time (*p* ≤ 0.05).
